# The effect of an economic empowerment and relationship strengthening intervention on food insecurity among couples living with HIV in Malawi

**DOI:** 10.1186/s12889-025-23911-w

**Published:** 2025-08-20

**Authors:** Joshua Kiyingi, Fred M. Ssewamala, Torsten B. Neilands, Scott Tebbetts, Nancy Mulauzi, James Mkandawire, Amy A. Conroy

**Affiliations:** 1 Department of Medicine, University of California San Francisco, San Francisco, CA USA; 2 Brown School, Washington University, St. Louis, MO USA; 3 Invest in Knowledge, Zomba, Malawi

**Keywords:** Food insecurity, HIV, Couples, Unhealthy alcohol use, Sub-Saharan Africa, Economic empowerment, Relationship strengthening

## Abstract

**Background:**

People living with HIV (PLHIV) are highly impacted by food insecurity through pathways including poor adherence to antiretroviral therapy and inadequate nutrition. Limited evidence exists on whether economic empowerment interventions can improve food insecurity among PLHIV in sub-Saharan Africa. We evaluated the effectiveness of *Mlambe*, an economic empowerment and relationship-strengthening intervention, on food insecurity among couples living with HIV who drink alcohol in Malawi.

**Methods:**

We analyzed data from 78 couples who participated in the *Mlambe* study, implemented in Zomba, Malawi. The study enrolled married couples living with HIV and unhealthy alcohol use (based on the AUDIT-C) from HIV care settings: an urban hospital, a rural private community, and a peri-urban health center. Couples were randomized into two groups: the *Mlambe* intervention or enhanced usual care (EUC). *Mlambe* intervention included incentivized savings accounts and sessions on financial literacy training and relationship skills over ten months. Study assessments occurred at baseline, 10- and 15-months. Food insecurity was assessed using the Household Food Insecurity Access Scale (HFIAS), which was categorized into four levels (food secure, mild, moderate, and severe food insecurity). We fit two-level, logistic mixed effects models testing the effect of *Mlambe* on severe food insecurity, given that most couples reported being food insecure.

**Results:**

The mean age of participants at baseline was 43.4 years, with 78.2% reporting primary education. On average, couples had been married for 13.8 years. Over half (53.3%) reported unhealthy alcohol use, 91.6% were HIV-positive, and 57.1% experienced severe food insecurity. Couples in *Mlambe* intervention showed a significant reduction in severe food insecurity as compared to the EUC arm at the 15-month follow-up (OR = 0.81, 95% CI: 0.66, 0.99). No significant reductions were observed at 10 months, which immediately followed the intervention period.

**Conclusion:**

*Mlambe* intervention demonstrated a significant reduction in severe food insecurity among HIV-positive couples with unhealthy alcohol use at 15-months. This pilot study provides evidence that integrated interventions targeting economic and relationship factors at household level have the potential to effectively reduce food insecurity in settings like Malawi. A full-scale efficacy study is needed to confirm findings with a larger sample and longer follow-up.

**Study clinical trial registration:**

*Mlambe* was registered on ClinicalTrials.gov (NCT #04906616, 08/15/2019).

## Background

Food insecurity is defined as the lack of consistent physical and economic access to sufficient food for a healthy and productive life [1, 2]. In many countries across Sub-Saharan Africa (SSA), food insecurity levels have been increasing significantly over time [3]. As of 2023, an estimated 900 million people in SSA experienced moderate or severe food insecurity, with over 342 million individuals facing severe food insecurity [4]. Food insecurity in SSA is driven by a confluence of factors, including climate variability and extreme weather events, poor macroeconomic performance, and insufficient government investment in food subsidy programs [4–8].

Food insecurity significantly impacts HIV/AIDS treatment outcomes [9, 10]. People living with HIV (PLHIV) who experience food insecurity often have poor adherence to antiretroviral therapy (ART), decreased engagement in medical care, poor mental health, and inadequate nutritional status [9–13]. These challenges contribute to worsened virologic and immunologic responses, ultimately increasing the risk of serious illnesses and mortality [14–18]. Conversely, HIV infection can worsen food insecurity by reducing economic productivity and weakening social support systems to offset food shortages [10, 19]. The stigma surrounding HIV may further isolate affected individuals and families from seeking food assistance, and the financial burden of medical expenses can deplete household resources, resulting in less money available for food [20].

Food insecurity is also associated with unhealthy alcohol use (positive screen with the Alcohol Use Disorders Identification Test Consumption (AUDIT-C)) [21]. In SSA, alcohol use is common, with alcohol dependence and prevalence reported at 9.5% and 4.3%, respectively [22]. Alcohol consumption can contribute to household poverty by diverting limited financial resources away from essential needs such as food. This economic strain, in turn, can negatively impact family relationships, undermine social support, and jeopardize overall well-being, affecting both individual and family health [23–25]. Similar to food insecurity, unhealthy alcohol consumption is associated with unsuppressed viral load, partly due to reduced medication adherence [24, 26–28]. Thus, the syndemic of food insecurity, alcohol use, and poverty further exacerbates underlying health challenges faced by PLHIV [14, 29].

Improvements in food security and poverty reduction are widely recognized as critical components of an effective global HIV response [10, 14, 30]. There is significant potential for economic-strengthening interventions to address food insecurity, alcohol use, and HIV. For example, in Kenya, an agricultural livelihood intervention that included a microfinance component led to improved food security and diet diversity among PLHIV [10]. Similarly, in Zimbabwe, a combined intervention of microfinance, life skills, health education, and vocational training improved food security among female orphans at risk of HIV [31]. Another observational study in Ethiopia comparing microfinance credit users to non-credit users found that credit users experienced improved food security and a significant increase in average daily calorie intake per capita over time compared to non-credit users [32]. In Uganda, the *Suubi* intervention studies, which implement economic empowerment (EE) interventions, have reported improved ART adherence among adolescents [33–35]. Additionally, in South Africa, a combined intervention of microfinance, gender training, and HIV risk reduction led to decreased HIV risk behaviors and increased uptake of voluntary counseling and testing for HIV among women [36, 37]. To date, to our knowledge, there are no economic empowerment interventions for unhealthy alcohol use that have evaluated impacts on food insecurity among PLHIV in SSA.

Economic interventions at the household level may be most effective when combined with relationship-strengthening (RS) interventions that improve couple dynamics, including communication around family finances and shared savings goals. For example, in Nigeria, a gender-transformative RS intervention aimed at enhancing women’s participation in household decision-making led to increased female involvement in financial decisions [38]. In the US, an RS intervention that integrated relationship and financial education workshops, along with referrals to community services, improved dyadic strategies for managing financial and non-financial stress among low-income African American couples [39]. Despite these promising findings, the impact of EE coupled with RS interventions on reducing food insecurity among PLHIV remains relatively unexplored, particularly among couples with co-occurring HIV and unhealthy alcohol use in SSA.

To bridge this gap, we designed and evaluated *Mlambe*, a behavioral intervention aimed at addressing unhealthy alcohol use among couples living with HIV in Malawi. *Mlambe*, a randomized control trial (RCT), integrates EE and RS activities by providing incentivized savings accounts, financial literacy education, and relationship skills training to improve partner support and communication, with a targeted emphasis on managing household finances, addressing alcohol consumption, and promoting HIV prevention [40]. *Mlambe* is grounded in the asset theory, which posits that economic asset accumulation may result in financial stability, increased hopefulness for the future, less risk-taking behaviors, and other social benefits [41]. Households and communities can develop by increasing savings and accumulating assets, but not income alone [42, 43]. *Mlambe* aims to accomplish this by providing matched savings accounts combined with financial literacy training and support for starting a household income-generating activity to build assets [40, 44]. This EE approach has been proven effective in other settings in SSA, including Uganda, Kenya, and Zimbabwe [31, 35, 45–47]. *Mlambe* also incorporates relationship skills training to teach couples how to collaborate around finances and work towards their saving goals as a family [40].

Using data from a pilot trial, the current study assessed the effect of the *Mlambe* intervention on reducing food insecurity as a secondary outcome among HIV-positive couples affected by unhealthy alcohol use in Malawi.

## Methods

### Study setting

Malawi is one of the poorest countries in SSA, with 71% of adults living on $2.15 USD or less per day [48]. Rates of food insecurity are also high in this country, with 9 million people, almost half the population, affected. Over 4 million are facing acute food insecurity, signifying conditions consistent with a humanitarian emergency [49]. This crisis is compounded by high levels of alcohol consumption and HIV. In Malawi, over 51% of men who consume alcohol exhibit patterns of unhealthy alcohol use [50]. This study was conducted in Zomba District, situated in the southern region of Malawi, which has an estimated population of nearly one million people [51]. The district faces a high HIV burden, with approximately 15% of adults living with the virus [52].

### Recruitment

The study recruited 78 couples in a period of six months from three different HIV care settings: an urban district hospital, a rural private community hospital, and a peri-urban public health center. The research team presented information about the study in HIV clinic waiting rooms during patient appointments. Interested patients could then approach recruiters for further information. For patients arriving after the information sessions, recruiters directly approached patients in the waiting rooms and explained the study. If interested and eligible, patients received an information card to share with their spouse, who could then contact the study team for a phone screening. Following partner screening, eligible couples were scheduled for their first appointment to obtain written, informed consent in Chichewa. The consenting process was conducted in private settings with each partner separate to ensure voluntary participation. A CONSORT diagram detailing recruitment and retention at each time point is published elsewhere [40].

### Inclusion and exclusion criteria

Eligibility criteria for the study included being in a non-polygamous marital or cohabiting union, aged 18 years or older, and having at least one partner having a positive screen on AUDIT-C (≥ 4 for men, ≥ 3 for women in the prior three months) and enrolled on ART for at least six months (designated as the “index patient”). Both partners living with HIV were required to have disclosed their HIV status to each other. Exclusion criteria included couples reporting severe intimate partner violence (IPV) within the past three months using the WHO domestic violence module [53], or those expressing safety concerns regarding participation in the study. However, no couples were ultimately excluded based on these criteria [40].

### Randomization

Couples recruited for the study were randomized into two groups: the *Mlambe* intervention and enhanced usual care (EUC). Block randomization with randomly permuted block sizes was employed using a secure, computer-generated process. To ensure balanced group sizes across sites, 78 couples were randomized in blocks of 20. Three blocks were comprised of couples recruited from the same clinic site, while the fourth block included a mix of couples from all three sites. Once each block of 20 couples was enrolled, a randomization ceremony was conducted. During this ceremony, each couple selected an envelope containing their assigned intervention group. More details are published elsewhere [40].

### The Mlambe intervention

The *Mlambe* intervention consisted of EE activities focused on incentivized savings accounts and financial literacy training, and RS activities focused on relationship skills education and couple communication training [24, 40, 44]. Ten monthly sessions were delivered by trained facilitators at community-based locations or HIV clinics. A structured manual was used to guide the delivery of all sessions. While most sessions were group-based (approximately 10 couples), two sessions were one-on-one counseling sessions where couples practiced relationship skills with a trained counselor. Each session lasted between 80 and 210 min. Session topics included the health and relational harms of alcohol use, strategies for reducing alcohol consumption, banking services, saving and asset building, debt management, relationship dynamics, gender-based power, and communication skills. The RS sessions encouraged participants to reflect on concepts of relationship power, emphasizing shared power and minimizing imbalances in power between partners. Sessions also focused on strengthening trust, intimacy, and communication, teaching couples how to express gratitude and provide meaningful support. Alcohol reduction messages and activities were included throughout the sessions; for example, budgeting sessions highlighted the financial burden of alcohol and alternative household expenditures. Couples also learned and practiced communication skills with a counselor to navigate challenging conversations about alcohol use and household economic conditions.

At the start of the intervention period, couples opened a joint banking account at a commercial bank and were eligible to receive a 1:1 match (up to $10 USD per month) for savings accumulated each month over the study period. Matched funds could be accessed for medical, educational, or expenses related to an income-generating activity (IGA). The couples would not receive matched funds if they withdrew money for reasons other than those stated above. In the final session, community extension workers provided guidance on achieving IGA business goals, such as livestock rearing or vegetable farming, and offered ongoing support. Upon completing the program, couples could withdraw their remaining savings (including matched funds) to invest in income-generating activities or small businesses. Receipt of the matched savings component was contingent upon attending at least eight out of the ten sessions. The program reported 100% attendance and high participant satisfaction with the sessions [24, 40]. Participants in *Mlambe* saved an average of $7.75 USD per month, with mean cumulative savings of $77.50 USD (range: $22–$194) before the match, and approximately $145 USD (range: $44–$292) after including the 1:1 matched contributions over the 10-month period [40].

In the EUC control group, usual care was based on Malawi’s Ministry of Health Guidelines for Clinical Management of HIV [54], and included monitoring alcohol-related non-adherence, treatment failure, and liver disease. Couples in the EUC arm also received 10–15 min of brief alcohol counseling based on the World Health Organization (WHO) intervention model [40]. Training procedures and fidelity assessments for the intervention are detailed in prior studies [44].

### Data collection and measurements

Data were collected at baseline, 10 months, and 15 months follow-up (post-intervention initiation) between June 2021 and July 2023. Data were collected using structured instruments administered by interviewers using REDCap^®^ on tablet devices. Instruments were administered in Chichewa, the local language used in the study region. The instruments were translated into Chichewa and back-translated to English for quality control purposes.

The outcome measure for the current study was food insecurity, assessed using the HFIAS [1]. The nine-item scale assesses the household’s experience of food insecurity with possible responses including *Never = 0*,* Rarely = 1*,* Sometimes = 2*, and *frequently = 3*. The reliability of the scale was 0.86, indicating high internal reliability. Given that most participants reported some level of food insecurity (mild: 5.7%, moderate: 24.4%, or severe: 57.1%), scale scores were dichotomized into two categories: *secure*,* mild*,* and moderate food insecurity = 0* and *severely food insecure = 1*. We also captured participants’ characteristics, including age in years, gender (male or female), education level (primary or less and post-primary), relationship duration in years, alcohol use using the AUDIT-C score [55], and number of days drinking based on the timeline follow-back [56].

### Data analysis

Data analysis was conducted using Stata version 18 [57]. Baseline participant characteristics are presented using means and standard deviations for continuous variables and frequencies and percentages for categorical variables. To test the effect of the *Mlambe* intervention on food insecurity, we fit a two-level multilevel logistic regression model. Separate sets of models were fit for predictions at 10-months and 15-months, controlling for baseline food insecurity value. Each model included fixed effects for the baseline food insecurity and treatment arm (intervention vs. control). Random intercepts were specified to account for the nested structure of the data, with individuals nested within dyads to address non-independence within couples. To explore potential moderation by baseline food insecurity value, a second series of logistic mixed-effects models were fit, including an interaction term between baseline food insecurity and treatment arm. All analyses used an intent-to-treat approach in which every couple was included with their original randomization group under the assumption that participants with incomplete data were missing at random (less than 5%).

## Results

Table 1 summarizes the sample characteristics at baseline for all participants. Participants had a mean age of 43.4 years (SD = 10.2), with 78.2% reporting primary education or less. On average, participants had been in their current relationship for 13.8 years (SD = 10.7). Over half of the participants (53.2%) reported alcohol use, with an average of 3.5 drinking days per month (SD = 5.6). The majority (91.7%) were HIV-positive, and 57.1% experienced severe food insecurity. There were no significant differences in severe food insecurity by treatment arm at baseline.

**Table 1:** Baseline characteristics of the Mlambe sample (*N* = 156 individuals)

Variable	Total Sample	EUC arm	Mlambe arm	*p*-value
	*N*(%), Mean(SD)	*N*(%), Mean(SD)	*N*(%), Mean(SD)	t-test/χ^2^(df)
Sample	*N* = 156	*N* = 78 (50.0%)	*N* = 78 (50.0%)	
Age in years (min/max: 21–80)	43.4 (10.2)	43.7 (10.3)	43.24 (10.1)	0.80
Education				
Primary school or less	122 (78.2%)	60 (49.2%)	62 (50.8%)	0.70 (1)
Post-primary	34 (21.8%)	18 (52.9%)	16 (47.1%)	
Relationship duration (in years)	13.75 (10.7)	14.2 (12.1)	13.28 (9.2)	0.59
Alcohol use				
Abstainer	73 (46.8%)	38 (52.1%)	35 (47.9%)	0.63 (1)
Drinker	83 (53.2%)	40 (48.2%)	43 (51.8%)	
Number of drinking days (0–30)	3.48 (5.6)	3.4 (5.8)	3.56 (0.6)	0.85
HIV positive	143 (91.7%)	73 (51.1%)	70 (48.9%)	0.50 (1)
Food insecurity				
Secure/Mild/Moderate	67 (42.9%)	31 (46.3%)	36 (53.7%)	0.42 (1)
Severely Insecure	89 (57.1%)	47 (52.8%)	42 (47.2%)	

*df* degrees of freedom

Table 2 presents between-arm differences in severe food insecurity at baseline, 10-months, and 15-months. The *Mlambe* intervention had no significant effect on severe food insecurity at 10-months. However, *Mlambe* showed a significant reduction in severe food insecurity compared to the EUC arm (*p* < 0.05) at 15-months (OR = 0.81, 95% CI: 0.66, 0.99, *p* = 0.04). We tested for an interaction between baseline food insecurity level and treatment arm at the 15-month visit, however, these differences were not statistically significant.

**Table 2:** Between-arm differences in food insecurity (*N* = 156 individuals)

Severe food insecurity	Mlambe Arm	EUC Arm	OR	95% CI	*p*
	*N* (%)	*N* (%)			
Baseline	42 (47.2%)	47 (52.8%)	0.77	0.41, 1.45	0.42
10-months	64 (50.4%)	63 (49.6%)	1.03	0.83, 1.28	0.81
15-months	60 (46.2%)	70 (53.9%)	0.81	0.66, 0.99	**0.04**

The 10−month and 15−month models controlled for baseline food insecurity

Bold values indicate statistical significance at *p* < 0.05

## Discussion

This study examined the effect of a pilot study focused economic and relationship-strengthening intervention called *Mlambe* on the reduction of food insecurity among HIV-positive couples reporting unhealthy alcohol use in Malawi. We found that *Mlambe* was effective in reducing severe food insecurity at the 15-month visit. As couples accumulated savings and subsequently invested savings into a family business, it may have fostered a sense of financial stability, consequently improving access to food. Furthermore, relationship-strengthening sessions likely enhanced communication between partners, fostering improved collaboration around household finances and budgeting for food. This may have led to increased savings through reduced alcohol consumption and better management of household finances. These improvements may have contributed to the ability to consistently access and afford nutritious food. Our findings also align with other studies that have applied asset theory [41], upon which the *Mlambe* study is based.

Additionally, in many African settings, where men are often the primary breadwinners and decision-makers, coordination between spouses is critical to managing limited resources for food. Husbands typically provide income for household expenses such as food, while wives handle food purchasing and preparation, highlighting the shared responsibility in addressing household food insecurity [58]. The *Mlambe* pilot study contains sessions on gender-power dynamics and economic power, as well as communication skills to negotiate household purchases, including food. While we cannot assess which component played a stronger role in reducing food insecurity, it is important to acknowledge the unique contribution of *Mlambe* regarding the synergies between relationship skills and EE activities.

The nonsignificant findings at the 10-month follow-up could have reflected the sequence and timing of the program’s rollout activities. Couples primarily focused on maximizing matched savings during the 10-month intervention period while receiving FLT. After the 10-month period, couples invested the money saved plus the match in an IGA for up to 15 months. Given that the 10-month assessment occurred immediately after the intervention sessions and before couples started their IGAs, the full impact of these savings on household food security might not have been fully realized. Over time, accumulated savings were invested in IGAs, such as agribusiness, leading to improved household food intake, as observed at the 15-month follow-up.

Our findings align with other family economic empowerment studies, which demonstrated improvements in food security over time. The *Shamba Maisha* RCT in Kenya implemented agricultural and microfinance interventions to improve HIV health outcomes among PLHIV reported increased food security and greater frequency of food consumption [10]. Similarly, an RCT conducted in Zimbabwe, which integrated microgrants with life skills and health education as well as vocational training intervention for female orphans, reported a significant reduction in food insecurity [31]. Additionally, an observational study in Ethiopia showed that microfinance credit users experienced greater improvements in food security compared to non-credit users [32]. Our findings extend this literature by demonstrating the potential of other economic empowerment interventions (i.e., matched savings and FLT) in reducing food insecurity over time among PLHIV, specifically couples with unhealthy alcohol use. The high prevalence of food insecurity in SSA, primarily driven by extreme poverty and exacerbated by climate change, necessitates evidence-based interventions to mitigate the impact of climate change [4]. Consequently, the desired health outcomes for HIV-positive individuals may not be achieved with the current levels of food insecurity in the region. Therefore, the development and implementation of evidence-based interventions aimed at improving food security, like *Mlambe*, are crucial for optimizing HIV clinical outcomes. For families affected by HIV, it may require the collaboration of both partners around household finances to effectively reduce food insecurity and its detrimental effects on HIV-related health. *Mlambe* is the first intervention to integrate economic empowerment and relationship-strengthening strategies, uniquely addressing the needs of HIV-positive couples facing food insecurity and unhealthy alcohol use.

### Limitations

First, *Mlambe* was a pilot study with a small sample size. Pilot studies primarily serve to refine study design and optimize recruitment, retention, and participant engagement before embarking on larger-scale trials. This iterative process is crucial for ensuring the feasibility and effectiveness of future interventions [59–63]. Given the pilot nature, larger efficacy studies are needed to confirm the findings of this small trial. Second, food insecurity was originally classified into four levels: secure, mild, moderate, and severe; however, given the distribution of food insecurity in the sample, categories were collapsed into two groups: severe and non-severe food insecurity. Larger studies with different types of couples are needed to better understand the impacts on mild and moderate food insecurity. Moreover, food insecurity measures rely on self-reports, which might be influenced by social expectancies and recall biases. Future studies may incorporate other measures of diet diversity and quality, food frequency, and household coping mechanisms in combination with global measures of food insecurity. Third, to better understand the impact of the Mlambe intervention on food insecurity among couples living with HIV, it would be valuable to compare their outcomes with those of HIV-negative couples. However, this analysis was not feasible in the current study due to the small sample size. Future research with a larger sample could explore this comparison more effectively. Despite the limitations of the pilot study, the *Mlambe* program demonstrated the potential for an economic and relationship-strengthening intervention to mitigate food insecurity among couples living with HIV. Full-scale efficacy trials with a larger sample size and extended follow-up period are needed to confirm these promising effects on food insecurity.

## Conclusion

Even in this small pilot study, the *Mlambe* intervention demonstrated a reduction in food insecurity among HIV-positive couples affected by unhealthy alcohol use in Malawi. This intervention provides evidence that economic empowerment interventions coupled with relationship strengthening at the household level have the potential to reduce household food insecurity in SSA in the long term. Further research with larger sample sizes and longer follow-up periods is warranted to fully explore the long-term impact of these interventions on food security and overall health outcomes among couples living with HIV.

## Data Availability

The datasets generated and/or analyzed during the current study are not publicly available due to their sensitivity but are available from the corresponding author upon reasonable request.

## Abbreviations

ART: Antiretroviral therapy
AUDIT-C: Alcohol use disorders identification test consumption
EE: Economic empowerment
EUC: Enhanced usual care
FLT: Financial literacy training
HIV: Human immunodeficiency virus
IGA: Income-generating activity
IPV: Intimate partner violence
PLHIV: People living with HIV
RCT: Randomized controlled trials
RS: Relationship strengthening
SSA: Sub-Saharan Africa
WHO: World Health Organization
